# Psychopathy and Decision-Making: Antisocial Factor Associated With Risky Decision-Making in Offenders

**DOI:** 10.3389/fpsyg.2019.00166

**Published:** 2019-02-01

**Authors:** Xiaoqiang Yao, Fenghua Zhang, Tingting Yang, Tao Lin, Ling Xiang, Fuming Xu, Guanrui He

**Affiliations:** ^1^School of Psychology, Jiangxi Normal University, Nanchang, China; ^2^Key Laboratory of Psychology and Cognition Science of Jiangxi, Jiangxi Normal University, Nanchang, China; ^3^Nanchang Prison, Nanchang, China

**Keywords:** psychopathy, antisocial, decision making, general intelligence, Iowa Gambling Task, Game of Dice Task, offenders

## Abstract

Psychopathy is a personality development disorder increasing the risk of antisocial behavior. Studies on the relationship between psychopathy and decision-making have received limited attention and the result of studies is mixed. A present study examines whether or not the different factors of psychopathy are related to decision-making under risk and ambiguity in offenders and how they are related. Also, the study investigates whether general intelligence is associated with decision-making or moderates the relationship between psychopathy and decision-making. The results showed that only antisocial factor of psychopathy significantly correlates with Game of Dice Task (GDT) risky selections, but there no general relation between psychopathy and Iowa Gambling Task (IGT) performance. Lastly, general intelligence neither is related to decision-making under risk and ambiguity nor moderates the relationship between decision-making and psychopathy. The study results show that antisocial factor of psychopathy was associated with decision-making under risk rather than ambiguity. Our results also suggest that the antisocial factor of psychopathy was more related to executive dysfunction in offenders.

## Introduction

Psychopathy is a personality developmental disorder that is characterized by a constellation of interpersonally, affective, and behavioral features (Cleckley, 1941; Hare, 2003). Interpersonally, psychopaths are superficial, arrogant, and manipulative; affectively, they have shallow affect and display lack guilt, or remorse, and behaviorally, they are impulsive, risk-taking, and irresponsible (Hare, 1998). Psychopathy has been regarded as the “most important clinical construct in criminal justice system” (Hare, 1998). For psychopathy its strong predictive validity for institutional adjustment and recidivism (Walters, 2003). Previous study has showed that psychopathy is associated with a heightened propensity for violent behavior (Hare et al., 2000). In terms of recidivism, individuals with psychopathy are approximately three times more likely to reoffend than non-psychopaths (Hemphill et al., 1998). Therefore, it is an important goal to understand the nature of psychopathic cognitive impairment in forensic and criminal justice system.

### Psychopathy Assessment

The Psychopathy Checklist-Revised (PCL-R) was developed to assess psychopathy construct as described by Cleckley in forensic samples (Hare, 1991, 2003). In the last several decades, the PCL-R has been as one of the most widely means of assessing psychopathy. The PCL-R is a clinical rating scale consisting of 20 items, whose score is based on information collected from prison files and semi-structured interview. The factor analysis suggested that PCL-R contains two factors: Factor 1 (divisible into affective and interpersonal facets) and Factor 2 (divisible into lifestyle and antisocial facets). However, PCL-R has its limitations. First, the semi-structured interview is time-consuming, and cannot be administered in groups at same time. Second, training is required in the process of administered and score. Last but not least, the file records required for score are not always available.

Self-report measures for psychopathy have been developed to shake off these limitations (Levenson et al., 1995; Lilienfeld and Andrews, 1996). One of self-report scales suggested that promising measure of psychopathy is Levenson Self-Report Psychopathy Scale (LSRP; Levenson et al., 1995). The LSRP, which consists 26 items, can be divided into two separate scales: primary and secondary psychopathy. The primary psychopathy was created to assess selfish, manipulative posture toward others, and the secondary psychopathy was designed to assess impulsivity and self-defeating life style (Levenson et al., 1995). The LSRP has been found to correlate significant with both factors of PCL-R (Brinkley et al., 2001). While, Brinkley et al. (2008) was proposed a three-factor structure (egocentricity, callous, and antisocial) fit the data better than two-factor structure of LSRP in a female inmate’s sample. Subsequently, several studies have strongly given support for the three-factor model in offender, undergraduate, and community adult samples (Sellbom, 2011; Somma et al., 2014). In the cultural context of China, the three-factor structure among male offenders and college student is also confirmed (Shou et al., 2017; Wang et al., 2018).

### Psychopathy and Decision-Making

Abnormalities in brain regions of psychopaths are found in a several of studies. Gao et al. (2009) find cognitive and affective-emotional processing deficits to be associated with brain abnormalities, especially with the cases of structural function impairments in orbitofrontal, ventromedial prefrontal, and amygdala, which do exist in people with suffering from psychopathy. A review has revealed that psychopathy disorder is correlated with dysfunction in orbitofrontal limbic, anterior cingulate-orbitofrontal, and prefrontal-temporal-limbic networks (Del Casale et al., 2015). The ventromedial prefrontal cortex (vmPFC) plays a crucial role in affective decision-making processes, response reversal and response inhibition (Wallis, 2007). Prior research on psychopathy and decision-making suggest that reversal learning is impaired for highly psychopathic individuals (Blair, 2008; Finger et al., 2008). The somatic marker hypothesis posits that “somatic marker” biasing signals from body are represented and regulate decision-making in situations of complexity and uncertainly (Damasio, 1994, 1996). The theory points that “somatic markers” is to be processed in the vmPFC and amygdala. The empirical support for somatic marker hypothesis is based on findings from the Iowa Gambling task (IGT; Bechara et al., 1994; Bechara and Damasio, 2005).

The IGT, which is the act of replicating decision-making of real life, was developed by Bechara et al. (1994). The task of IGT requires participants to make series of selections from four decks of cards. Out of these four decks, two decks are assumed as being disadvantageous and distribute high immediate rewards with long-term loss, while the other two decks are assumed to be advantageous and provide lower immediate rewards and long-term profit. Some prior studies indicated patients with lesion from ventromedial and amygdala would select from disadvantageous decks because of their difficulties to anticipation of future consequence (Bechara et al., 1994, 1999).

Also, research on psychopathy with IGT has received limited attention and has been less conclusive. For example, Boulanger et al. (2008) observe that psychopathic individuals are characterized by impairment for decision-making compared with controls. Likewise, Mitchell et al. (2002) suggest that psychopathic offenders show a global tendency to choose disadvantageously compared with controls. Schmitt et al. (1999) in their study, as an example different with previous study results, compare psychopathic offenders with non-psychopathic offenders’ performance on IGT, but observe no group difference. The inconsistencies in the results of these studies could be explained by factors, such as differences of the samples selected.

Psychopathy as multi-dimensional construct is likely to have different relations with decision-making in different dimensions (Hughes et al., 2016). For example, Beszterczey et al. (2013) observed that only PCL-R factor 2 (lifestyle and antisocial) is significantly and negatively correlated with net score in ex-offenders, especially in block 4 and 5. Dean et al. (2013) demonstrate that only secondary psychopathy is associated with IGT risk choice in a college sample. However, Lösel and Schmucker (2004) find that there is no general relationship between psychopathy and IGT performance. Bass and Nussbaum (2010) find that only PCL-R facet 4 (antisocial facet) is significantly positively related with IGT scores in psychiatric inpatients. Similarly, Hughes et al. (2015) observe that higher levels of psychopathy are associated with more advantageous choices in incarcerated offenders, and higher levels of facet 4 can predict advantageous choice during the IGT learning phase. However, in Hughes et al. (2015) study, they told to participants that some decks may be better than others. Together, these studies provide mixed evidence for the relationship between psychopathy facet or factor and IGT performances.

In the IGT, the probabilities of outcome are unknowingly, this type of decision-making is regarded as a decision-making under ambiguity (Bechara and Martin, 2004). On the other hand, when the outcome probabilities of winning or losing are explicit, this type of decision is commonly referred to as a decision under risk (Brand et al., 2006). Most studies investigating decision-making under risk situations usually used either the Cambridge Gambling task (CGT) or Game of Dice Task (GDT; Brand et al., 2005; Rogers et al., 1999a,b). In the CGT task, a row of 10 red and blue boxes are presented to the participants. Under instruction, participants would decide and bet on whether a token has been hidden under a red or blue box. Actually, outcome probabilities of winning or losing associated with specific ratio of red to blue boxes in each trial. Previous study has suggested that vmPFC and insula play necessary roles in CGT task (Clark et al., 2008). Recently, Sutherland and Fishbein (2017) observed that higher levels of psychopathy indicate make more risky selections in CGT.

In the GDT, participants are asked to guess a number or combination of numbers (two, three, or four numbers) before rolling a dice. Each choice is associated with specific fictive gains and losses. If participants choose a single number, they only have a one in six chance to win 1000€. If the participants choose two numbers, they have a one in three chance to win 500€. Participants are also allowed to choose three numbers for a one in two chance to win 200€, or they can choose four numbers with two in three chance to win 100€. Neuroimaging study found that medial orbitofrontal cortex, ventral and dorsal striatum are activated in during GDT decision-making (Wilbertz et al., 2012). According to our best knowledge, there is no research to investigate the relationship between GDT performance and psychopathy. Svaldi et al. (2012) found female individuals with borderline personality disorder make risky decision significantly more frequent than the control group.

As we have discussed above, most current studies have examined psychopathy and decision-making under uncertainly by IGT, with little attention paid to decision under risk. Therefore, it is necessary to investigate whether or not different factors of psychopathy relate to decision-making under ambiguity and risk (Hughes et al., 2016).

### Intelligence and Decision-Making

The research on the intelligence and decision-making adds to the evidence that intelligence is a factor that may influence decision-making. For example, Deakin et al. (2004) found that intelligence modulates risk-taking during decision-making. Recently, a longitudinal study suggested intelligence positively predicted IGT performance and was a significant predictor for risk, but not ambiguity in adolescent (Almy et al., 2017). Moreover, IGT performance is also correlated with intelligence in both psychopathic individuals and cocaine-dependent patients (Monterosso et al., 2001; Mahmut et al., 2008).

However, inconsistent results have emerged in two studies that intelligence is not associated with IGT performance in psychopathy individual (Blair et al., 2001; Lösel and Schmucker, 2004). Lösel and Schmucker (2004) also tested whether intelligence moderates the relation between psychopathy and IGT, and the result has shown no moderation effect of intelligence between them. Taking the above studies together, it is still uncertain whether intelligence is related to decision-making or as a factor to explain these inconsistent findings from psychopathy and decision-making.

### The Current Study

There are two purposes in the current study: (1) to investigate the relationship between psychopathic factors and decision-making by different tasks and (2) to examine whether intelligence is related to decision-making, or as a moderator variable in psychopathy and decision-making. We predicted that there is no relation between psychopathy and IGT performance based on prior studies in male offenders (Schmitt et al., 1999; Lösel and Schmucker, 2004). Prior literature suggests that psychopathy trait is associated with risky selections in CGT (Sutherland and Fishbein, 2017). In terms of specific psychopathy factors or facet, research have found factor 2 or antisocial facet is associated with risky decision-making (Beszterczey et al., 2013; Dean et al., 2013). As such, we anticipated that antisocial factor of psychopathy would be associated with GDT performance. Based on previous research, we expected that intelligence would not relate to IGT performance and psychopathy in male offenders (Lösel and Schmucker, 2004). It is still unclear whether intelligence would be associated with GDT performance in offenders or psychopathic individuals.

## Materials and Methods

### Participants

Sixty-five male adult offenders from a domestic prison were volunteered for this study. Five participants were excluded from the final analysis, three participants were removed due to their continuous selection of the same deck in the IGT task and other two participants due to select the same answer in LSRP more than 10 consecutive times. The average age of participants was 39.82 years (SD = 8.59; range = 22–59) in the sample. Considering that there is a large age range of the participants in the current study, we conducted a Person correlation analysis between participants’ age and decision-making performance (both IGT and GDT). The results suggested that there is no significant correlation in participants’ age, IGT total net score (*r* = 0.03; *p* > 0.05), and GDT net score (*r* = −0.02; *p* > 0.05). Their offenses included drug trafficking (19.6%), property offenses (32.1%), and violent offenses (32.1%) and others. Demographic characteristics of the participants (*N* = 60) are presented in Table 1. Using G-Power (version 3.1; Faul et al., 2009), we found that 35 participants would ensure 80% statistical power in case of a large effect (Cohen’s *f*^2^ = 0.35) with three predictors and 0.05 Type I error rate in a prior analysis. All participants did not receive material or monetary rewards in the current study. Instead they can get educational reform scores by taking psychological tests or lectures, and they will get the corresponding educational reform scores based on the performance.

**Table 1:** Description of demographic data of sample.

	*N*	%
Race		
Han	55	91.7
Other	5	8.3
Education (highest degree obtained)		
Primary school	16	26.7
Junior high school	36	60.0
High school	5	8.3
College degree	3	5.0
Marital status (current)		
Unmarried	22	36.7
Married	29	48.3
Divorced	9	15.0

All participants gave their written informed consent prior to the present study. This study has been approved and performed in accordance with the guidelines for ethics committees at both Nanchang Prison and Jiangxi Normal University.

### Materials

#### Iowa Gambling Task (IGT; Bechara et al., 1994)

**FIGURE 1:**
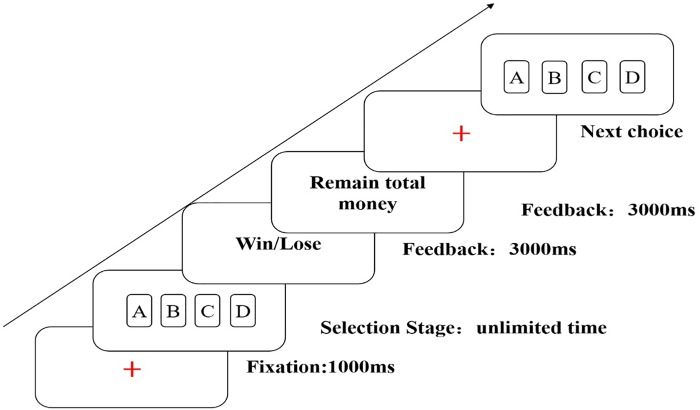
An example of a choice in the Iowa Gambling Task (IGT).

We used a modified version of the IGT to measure decision-making under ambiguity. Consistent with the design of the classic IGT, the task involves making 100 selections (five blocks of 20 selections) from four decks of cards (A, B, C, and D). Decks A and B are disadvantageous: higher reward (an average gain of 100 points for each win) but higher future losses (−250 point pre-10 cards). Decks C and D are advantageous: lower reward (an average gain of 50 point for each win) but lower future losses. At beginning of the task, we will give a loan of 2000 point. In each trial of the task, participants will first see a point of fixation with a duration of 1000 ms, and then choose one card from four decks of card, which would result in win or loss. Participants see the updated total scores cumulative points for 3000 ms on the screen after each choice (see Figure 1). If participants could not understand the task, we will add another five choices for practice. The results of the practice are not a part of final results. The participants were instructed to earn more and more points.

#### Game of Dice Task (GDT; Brand et al., 2005)

**FIGURE 2:**
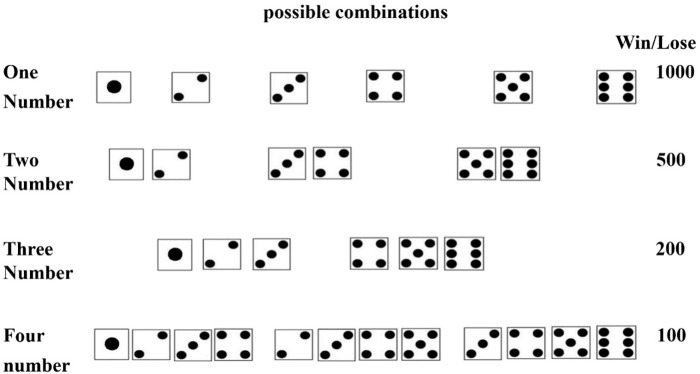
The possible die combinations associated with each option in the Game of Dice Task (GDT).

We used a modified version of the GDT to measure decision-making under risk. The GDT is a decision-making task that explicitly provides information about the rules for winning and losing associated with a given choice. At the beginning of the task, we will give a loan of 2000 point and have to play 32 trials. The GDT was required to throw a single virtual dice, and the four options represent the number of dice combinations for participants to bet on. Options “1,” “2,” “3,”and “4” represent the four combinations of dice, respectively. Participants can choose option “1” having a probability of 1:6 to win 1000 point. Participants choosing option “2” have a probability of 1:3 to win 500 points. A further option is to choose “3,” by which participants have a probability of 1:2 to win 200 points. Lastly, participants may also choose option “4” having a probability of 2:3 to win 100 points (see Figure 2). The options “1” and “2” are defined as high-risk choices as they the probability to win is less than 34%. The other two options are defined as low-risk choice as they have a win probability of 50% or higher. Before beginning, participants are explicitly informed about the rules for winning and losing, and that amount of point is associated with each of the options chosen. Like the IGT, there are three practice trials in the GDT. The results of the practice are not included in the final results. The participants were instructed to win as much point as possible.

#### Levenson Self-Report Psychopathy Scale (LSRP; Levenson et al., 1995)

The LSRP is a 26-items self-report questionnaire rated on a four-point Likert scale ranging from strongly disagree to strongly agree. A three-factor structure of LSRP using 19 of the original 26 items contains three subscales, Egocentricity, Callousness, and Antisocial. The Egocentricity scale is comprised of 10 items, the Callousness 4 items, and Antisocial 5 items. We used a Chinese version LSRP scale, which was translated by Shou et al. (2017). Cronbach’s alpha for these scales was Egocentricity = 0.70, Callousness = 0.64, Antisocial = 0.63, and 19 items = 0.80. In the current study, the Cronbach’s alpha coefficients for Callousness and Antisocial scale are a bit low. However, Clark and Watson (1995) have argued that reliabilities between 0.60 and 0.70 may be adequate, especially for those scales with small numbers of items. Moreover, Clark and Watson (1995) have recommended that average inter-item correlations may be a more useful way measuring internal consistency. They have argued that the average inter-item correlations fall in the range of 0.15–0.50. In the current study, the average inter-item correlations for three factors were Egocentricity = 0.19, Callousness = 0.23, and Antisocial = 0.25.

#### Raven’s Advanced Progressive Matrices (RAPM; Raven and Court, 1998)

In the current study, we used a RAPM Set 1 as measure of general intelligence. This test consists 12 items of increasing difficulty. Scores were calculated by summing the number of correct items. Descriptive statistics of LSRP and RAPM can be found in Table 2.

**Table 2:** Descriptive statistics for the RAPM Set 1, LSRP, IGT, and GDT net score (*n* = 60).

	*M ± SD*
RAPM Set 1	3.57 ± 3.43
Egocentricity	20.82 ± 4.56
Callousness	6.98 ± 2.30
Antisocial	10.60 ± 2.76
IGT block 1 net score	−1.80 ± 7.03
IGT block 2 net score	−2.30 ± 7.38
IGT block 3 net score	−2.20 ± 7.29
IGT block 4 net score	−1.43 ± 8.55
IGT block 5 net score	0.30 ± 8.53
IGT total net score	−7.43 ± 22.09
GDT net score	3.03 ± 20.74

*Note: M ± SD = mean ± standard deviation; RAPM Set 1 = Raven’s Advanced Progressive Matrices; IGT Net score = (C + D) − (A + B); GDT net score = (3 + 4) − (1 + 2).*

### Procedure

The whole experiment was conducted in two quiet and appropriate temperature rooms and was divided into two parts. First, participants were requested to complete a battery of self-report questionnaire individually in a room. Then participants were requested to complete the IGT and GDT task in other room after first part. Half of the participants completed the IGT first and then completed the GDT, while the others did in opposite order. There was a break of 15 min for rest between the self-report questionnaires and decision-making task. The whole experiment lasted for 1 h. The two decision-making tasks were presented on the computer using E-Prime2.0 software package (Psychology Software Tools, Pittsburgh, PA, United States).

### Statistical Analyses

When we analyzed IGT performance, a net score was calculated by subtracting the numbers of disadvantageous choice (A and B) from the numbers of advantageous choice (C and D) in each block of 20 cards. The total net score of IGT is the sum of net scores of five blocks. Regarding the GDT, we calculated a net score by subtracting the number of high-risk choices (1 and 2) from the number of low-risk choices (3 and 4), such that a higher net score indicates non-risky performance. Moderation analysis using Hayes (2013) PROCESS macros for SPSS, has applied 1000 bootstrapping samples with 95% bias-corrected confidence intervals (CIs). An alpha level of 0.05 was utilized for all analyses. Statistical analyses were conducted with SPSS 20.0 (IBM Corp., United States).

## Results

### Psychopathy, IGT Performance, and General Intelligence

Correlation analyses were carried out among psychopathy, IGT performance, and intelligence (see Table 3). We found Egocentricity was related to Callousness (*r* = 0.33; *p* < 0.001) and Antisocial (*r* = 0.59; *p* < 0.001). The results also showed that Egocentricity, Callousness, and Antisocial were not related to the net score across blocks of IGT task and total net scores. Lastly, there were no significant correlations between IGT and intelligence.

**Table 3:** Correlations between RAPM Set 1, psychopathy, and IGT performance.

	1	2	3	4	5	6	7	8	9	10
1 RAPM Set 1	–									
2 Egocentricity	−0.13	–								
3 Callousness	−0.19	0.33^∗^	–							
4 Antisocial	−0.12	0.59^∗∗^	0.23	–						
5 IGT block 1 net score	−0.05	−0.12	0.04	−0.12	–					
5 IGT block 2 net score	−0.11	0.05	−0.08	−0.04	0.04	–				
6 IGT block 3 net score	−0.02	−0.00	−0.03	−0.16	0.05	0.43^∗∗^	–			
7 IGT block 4 net score	−0.03	−0.06	−0.16	−0.09	0.05	0.21	0.49^∗∗^	–		
8 IGT block 5 net score	0.05	0.15	0.13	0.11	0.02	−0.26^∗^	0.01	0.43^∗∗^	–	
9 IGT total net score	−0.05	0.01	−0.04	−0.10	0.38^∗∗^	0.47^∗∗^	0.69^∗∗^	0.81^∗∗^	0.48^∗∗^	–

*Note: ^∗∗^p < 0.01, ^∗^p < 0.05, two tailed.*

### Psychopathy, GDT Performance, and General Intelligence

The correlations between GDT performance and psychopathy were also analyzed (see Table 4). The results indicated that only Antisocial factor was negatively correlated with GDT net score (*r* = −0.35; *p* < 0.01). General intelligence was not associated with GDT performance.

**Table 4:** Correlations between GDT performance, RAPM Set 1, and psychopathy.

	1	2	3	4	5
1RAPM Set 1	–				
2 Egocentricity	0.13	–			
3 Callousness	0.19	0.33^∗^	–		
4 Antisocial	0.12	0.59^∗∗^	0.23	–	
5 GDT net score	0.05	0.13	0.18	−0.35^∗∗^	–

*Note: ^∗∗^p < 0.01, ^∗^p < 0.05, two tailed.*

### Regression Analyses

A multiple regression using standard or simultaneous entry was used to evaluate whether participant’s psychopathy can significantly predict GDT performance. The results also suggested that only antisocial factor was significantly related to GDT net score [standardized beta = −0.415; *t* = −2.72; *p* < 0.01; *F*(3,56) = 3.29; *p* < 0.05; adjusted R square = 0.104].

### Moderation Analysis

We conducted a series moderation analysis to test whether intelligence moderated the relationships between psychopathy factors and decision-making performance. In the GDT, the three factors of psychopathy are as independent variable separately, GDT net score as dependent variable and intelligence as moderator variable. When Egocentricity as an independent variable, the interactions effect between is not significant between Egocentricity (*β* = 0.07;95%CI [−0.282,0.419]), and so is Callousness (*β* = 0.11;95%CI [−0.774,0.998]). Lastly, when the Antisocial is regarded as an independent variable, the interaction effect between Antisocial and intelligence is also not significant (*β* = 0.07;95%CI [−0.346,0.491]).

In the IGT, three moderation analysis was also performed to test the interaction effect between psychopathy and intelligence. When the independent variable is Egocentricity, the interaction effect is non-significant between Egocentricity and intelligence (*β* = −0.21;95%CI [−0.717,0.305]), and so is Callousness (*β* = 0.23;95%CI [−1.200,1.650]). Finally, when the independent variable is antisocial, the interaction between intelligence and antisocial was not significant (*β* = −0.31;95%CI [−1.075,0.457]).

## Discussion

The current study aimed to examine the relationship between decision-making and different psychopathy factors in an offender sample. We also investigated whether general intelligence is related to risk-taking or moderated the relationship between risk-taking and psychopathy. The results revealed that there is no general relation between psychopathy and IGT performance, only antisocial factor of psychopathy is associated with risky decision-making on the GDT. General intelligence is not related to decision-making and not moderated the relationship between psychopathy and decision-making performance.

The present study is failure to indicate any relationships between IGT and psychopathy, which is just in line with the previous results (Schmitt et al., 1999; Lösel and Schmucker, 2004). However, this finding is contrary to previous studies that factor 2 or secondary psychopathy was related to IGT performances (Beszterczey et al., 2013; Dean et al., 2013). We suspect that the discrepancy results may be caused by differences in the sample and psychopathy measures. The sample in Dean et al. (2013) study is college student, and psychopathy measured via LSRP. In another study, Beszterczey et al. (2013) had chosen a sample of ex-offenders and measured psychopathy by PCL-R. The current study and other two studies are all male incarcerated male offenders (Schmitt et al., 1999; Lösel and Schmucker, 2004). On the other hand, it has been claimed that participants’ card selections 41–100 in IGT may be classified as being the decision-making under risk (Noël et al., 2007; Sinz et al., 2008). Dean et al. (2013) found that during the stabilization phase (blocks 3–5) secondary psychopathy was positively associated with risky deck selections. Similarly, factor 2 of PCL-R related to IGT block 4 and block 5 has been observed in another study (Beszterczey et al., 2013). Taken together, the two studies found that factor 2 and secondary psychopathy was associated with decision-making under risk. Although our results revealed no relation between psychopathy and IGT performance, we found a correlation between antisocial factor of psychopathy and GDT risk-taking.

Correlations only existed between Antisocial and GDT risky decision-making. To our best knowledge, no studies have examined the relationship between GDT performance and psychopathy. The finding in this study was quite similar to that by Sutherland and Fishbein (2017). They found that highly level of psychopathy (LSRP) was associated with great tendencies to make risky selections in the context of risky decision-making. Prior literature has suggested that higher scores on the antisocial factor of LSRP were associated with past antisocial behavior, self-reported hostility, substance abuse, and aggression (Brinkley et al., 2008). Indeed, Brand et al. (2005) reported that GDT performance may be mediated by subcomponents of executive functions. Moreover, Ross et al. (2007) suggest that secondary psychopathy was positively predictive of symptoms of executive function. Previous study factor analysis revealed that antisocial factor appears to correspond to the secondary psychopathy (Brinkley et al., 2008). Taken together, these findings suggested that individuals with antisocial psychopathic features tend to make risky selections on the GDT may associate with executive dysfunction. Lastly, we found that only antisocial factor is related to risk decision-making, but not related to decision-making under ambiguity. This finding also supports the view that risk decision-making and ambiguous decision-making have distinct neural processes (Brand et al., 2006; Brand and Altstötter-Gleich, 2008).

The results of correlation and moderation analysis suggested that general intelligence was not correlated with decision-making performance or moderated the relationship between psychopathy and decision-making, which is consistent with other study results. For example, Lösel and Schmucker (2004) found that intelligence was not related to IGT performances in a male offender sample. However, this finding is contrary to previous studies (Monterosso et al., 2001; Deakin et al., 2004; Mahmut et al., 2008; Almy et al., 2017). This inconsistency may be caused by different measures and samples selected. For example, Mahmut et al. (2008) choose a sample of college student and measure intelligence by Wechsler test.

It is acknowledged that there are limitations in the study. First, what one should pay attention to is the lack of non-offender counterparts. Therefore, it is unclear whether offenders will exhibit any difference in decision-making under risk and ambiguous decision. Second, we did not measure other cognitive abilities, for example, the previous studies suggested that deficits in working memory have been shown to contribute to poor performances on the IGT and GDT (Bechara and Martin, 2004; Dretsch and Tipples, 2008; Schiebener et al., 2013). It is possible that the relationship between antisocial and GDT performances was moderated by working memory. However, with a small sample size, caution must be applied. Further study using a larger sample size for each group is needed.

To sum up, this study found that only antisocial factor of psychopathy was significantly related to GDT risky selections rather than IGT performances. Furthermore, intelligence has nothing to do with decision-making. In general, the results of this study indicate that antisocial factor of psychopathy is more related to cognitive dysfunction which and confirms the previous finding that antisocial factor is a better predictor of decision-making impairments. It also means that the decision-making is different under risk and ambiguity is different.

## Author Contributions

FZ and XY equally contributed to the design of the study. XY prepared the experimental materials, collected the data, analyzed the results, and wrote the manuscript. FZ. and TY contributed to experimental materials, participated to part of the data analysis, programmed the experiment, collected the data, and revised the writing of the manuscript. TL and GH collected the data. All seven authors reviewed the manuscript.

## Conflict of Interest Statement

The authors declare that the research was conducted in the absence of any commercial or financial relationships that could be construed as a potential conflict of interest.
